# Service provision and barriers to care for homeless people with mental health problems across 14 European capital cities

**DOI:** 10.1186/1472-6963-12-222

**Published:** 2012-07-27

**Authors:** Réamonn Canavan, Margaret M Barry, Aleksandra Matanov, Henrique Barros, Edina Gabor, Tim Greacen, Petra Holcnerová, Ulrike Kluge, Pablo Nicaise, Jacek Moskalewicz, José Manuel Díaz-Olalla, Christa Straßmayr, Aart H Schene, Joaquim J F Soares, Andrea Gaddini, Stefan Priebe

**Affiliations:** 1 Health Promotion Research Centre, National University of Ireland Galway, University Road, Galway, Ireland; 2 Unit for Social and Community Psychiatry, Queen Mary University of London, Mile End Road, London, UK; 3 Department of Hygiene and Epidemiology, University of Porto Medical School, Al Prof Hernani Monteiro, 4200-319, Porto, Portugal; 4 National Institute for Health Development, 1096 Budapest Nagyvárad tér 2, Budapest, Hungary; 5 L Laboratoire de recherche, Etablissement public de santé Maison Blanche, 3-5 rue Lespagnol, 75020, Paris, France; 6 Department of Psychiatry, 1st Faculty of Medicine, Charles University, Ke Karlovu 11/120 00, Prague, Czech Republic; 7 Clinic for Psychiatry and Psychotherapy, Charité, University Medicine Berlin, CCM, Charitéplatz 1, 10117, Berlin, Germany; 8 Institute of Health and Society (IRSS), Université Catholique de Louvain, Clos Chapelle-aux-Champs, 30.05 B-1200, Bruxelles, Belgium; 9 Institute of Psychiatry and Neurology, ul. Sobieskiego 9, 02-957, Warsaw, Poland; 10 Ma Madrid Salud, Calle Juan Esplandiú n 13, 28007, Madrid, Spain; 11 Ludwig Boltzmann Institute for Social Psychiatry, Lazarettgasse 14A-912, 1090, Vienna, Austria; 12 Academic Medical Center, University of Amsterdam, Meibergdreef 5, Room PA1-156, 1105 AZ, Amsterdam, The Netherlands; 13 Department of Public Health Sciences, Karolinska Institute, Norrbacka, SE-171 76, Stockholm, Sweden; 14 Laziosanità ASP - Public Health Agency, Lazio Region, Via di S. Costanza 53, 00198, Rome, Italy

## Abstract

**Background:**

Mental health problems are disproportionately higher amongst homeless people. Many barriers exist for homeless people with mental health problems in accessing treatment yet little research has been done on service provision and quality of care for this group. The aim of this paper is to assess current service provision and identify barriers to care for homeless people with mental health problems in 14 European capital cities.

**Method:**

Two methods of data collection were employed; (i) In two highly deprived areas in each of the 14 European capital cities, homeless-specific services providing mental health, social care or general health services were assessed. Data were obtained on service characteristics, staff and programmes provided. (ii) Semi-structured interviews were conducted in each area with experts in mental health care provision for homeless people in order to determine the barriers to care and ways to overcome them.

**Results:**

Across the 14 capital cities, 111 homeless-specific services were assessed. Input from professionally qualified mental health staff was reported as low, as were levels of active outreach and case finding. Out-of-hours service provision appears inadequate and high levels of service exclusion criteria were evident. Prejudice in the services towards homeless people, a lack of co-ordination amongst services, and the difficulties homeless people face in obtaining health insurance were identified as major barriers to service provision.

**Conclusions:**

While there is variability in service provision across European capital cities, the reported barriers to service accessibility are common. Homeless-specific services are more responsive to the initial needs of homeless people with mental health problems, while generic services tend to be more conducive to long term care. Further research is needed to determine the effectiveness of different service delivery models, including the most effective coordination of homeless specific and generic services.

## Background

Mental health problems are higher amongst the homeless population than amongst the general population [1-7]. The more severe the level of homelessness the poorer the level of mental health [3,8,9]. Less than a third of homeless people with mental health problems receive treatment [3].

A permanent residence still represents one of the main requirements for registering with the health care systems in a number of European countries [10,11]. Homeless people are often reported as having problems registering with health services and often try and access mental health care through accident and emergency services, where it is unlikely they will receive the appropriate care and treatment [5,7].

Entitlement to health care for homeless people does not always mean access [10,11]. Limited accessibility is often due to factors such as opening hours, inflexible appointment procedures and location [7,11]. Homeless people may also encounter attitudinal barriers within services and there is often an unwillingness or difficulty in the health services to accommodate the multiple and complex needs presented by homeless people [10-12].

Despite the strong link between homelessness and mental health and the many barriers homeless people face in accessing health services, there is little information on the characteristics and quality of service provision for homeless people in Europe [12]. This paper draws on findings from the PROMO project (DG Sanco: 2007 – 2010), whose aim was to assess current service provision and quality of care across 14 European capital cities for people from the following socially marginalised groups who experience mental health problems: long-term unemployed, homeless, sex workers, refugees and asylum seekers, undocumented migrants and travellers. The findings specific to the homeless population are presented here.

## Methods

The PROMO project included the selection of two highly deprived areas in each of the 14 participating capital cities. Within these areas the study sought to: (i) obtain information on services providing care for homeless people with mental health problems; (ii) assess the overall quality of care for homeless people with mental health problems. The definition of homelessness used in this study comprised the first two categories of the existing ETHOS typology [13], i.e. roofless (people sleeping rough or in emergency accommodation) and houseless (people in temporary accommodation).

### Identification of research areas

A total of 28 highly deprived geographic areas were selected for the PROMO assessment (See Table 1). The focus was on highly deprived areas due to the tendency for marginalised groups to be concentrated in these areas. The areas were identified by using the relevant local indices of public health and social deprivation. The population size of each research area was originally planned to be between 80,000 and 150,000 inhabitants. However, some flexibility in population size was allowed in order to accommodate different local contexts relating to administrative boundaries and service catchment areas. Also, in some cases one or more areas were combined to achieve the target size.

**Table 1:** Target areas identified for assessment in 14 EU capital cities

**COUNTRY/CAPITAL**	**AREA 1**	**AREA 2**
**(census year)**	**(population)**	**(population)**
**Austria/Vienna (2008)**	District 16 94,735	District 20 82,369
**Belgium/Brussels (2007)**	Schaerbeek + St Josse 113,493+ 23,785	Molenbeek 81,632
**Czech Republic/Prague (2006)**	Praha 3 + Praha 7 69,939 + 39,425	Praha 8 100,255
**France/Paris (2006)**	Secteur Flandre psychiatric sector 102,387	La Courneuve + Aubervilliers in Seine Saint Denis 37,347 + 73,506
**Germany/Berlin (2006)**	Wedding (the sub area of “Schillerpark” removed) 123,191	Kreuzberg 147,798
**Hungary/Budapest (2001)**	District VIII. 81,787	District VII. and IX. 64,137+ 62,995
**Italy/Rome (2007)**	District 7 117,479	District 15146,090
**Ireland/Dublin (2006)**	Dublin North Central 126,572	Dublin West 134,020
**Netherlands/Amsterdam (2006)**	Bos en Lommer + De Baarsjes + Geuzenveld-Slotermeer 30,045 + 33,767+ 41,314	Amsterdam Zuid Oost 78,922
**Poland/Warsaw (2006)**	Praga Polnoc 73,207	Wola 142,025
**Portugal/Lisbon (2001)**	A group of smaller areas 85,177	Marvila + Santa Maria dos Oliváis 82,753
**Spain/Madrid (2006)**	Villaverde 146,859	Centro 149,797
Sweden/Stockholm (2010*)	Rinkeby-Kysta + Spånga-Tensta + Skarpnäk 45,500 + 36,000 + 40,000	Södermalm 118,000
**UK/London (2001)**	Hackney 202,824	Tower Hamlets 196,106

*Figures provided were updated during the project using 2010 data.

### Assessment of services

The PROMO assessment of services sought to assess all mental health, social care and general health services that potentially serve marginalised groups with mental health problems. While the assessment was focused on the selected deprived areas, services located outside these areas but used by people from the target groups from the areas were also assessed.

The focus of the assessment of services in this paper is on all mental health, social care and general health services which are directed specifically at homeless people. While any health service may potentially be a resource for homeless people, homeless people don’t tend to access the more generic health services for reasons outlined in the introduction e.g. accessibility, lack of health insurance, lack of outreach services etc. Data on provision in the generic health services is also presented, focussing on how such provision compares to that in the homeless specific services.

Services were coded as homeless specific based on service self-definition. The coding of each service was done by the researchers who carried out the interviews in their capital city. In a small number of cases it was not clear which marginalised group the service was aimed at or whether the service was group specific or generic. In such cases if 50% or more of the clients were estimated to be homeless the service was designated as a homeless specific service. Services were also classified as either mental health, social care or general health services. This distinction was once again based on service self-definition. In cases where it was not clear whether a service was mental health specific or generic, if 50% of clients were estimated to have a mental health problem the service was classified as a mental health service.

### Assessment of services tool

A structured questionnaire was developed for the assessment of services using an iterative process involving researchers from all participating cities. It was translated into the languages of the participating countries and three pilot interviews were carried out in each city. The PROMO tool was designed to assess the following aspects of service provision:

1. Provider and funding information

2. Characteristics of staff

3. Service accessibility

4. Characteristics of clients

5. Programmes provided to clients from target groups

6. Co-ordination with other services

7. Service evaluation

Services were identified according to available directories of services and information from relevant local practitioners. Information gathered during the interviews was used to consistently update the list of services. The assessments were carried out by PROMO researchers in each of the 14 capital cities, either face-to-face or over the phone, with either the manager of the service or a member of staff with the relevant knowledge.

### Overall quality of care for homeless people with mental health problems

In order to assess the overall quality of care semi-structured interviews with ‘experts’ in mental health care for homeless people were conducted, one in each research area (n = 28). The experts were identified during the assessment of services phase of the study where service managers were asked to identify suitable interviewees. The criteria for inclusion were a good knowledge of local service provision and professional experience of providing or facilitating access to mental health care for homeless people. If such an expert could not be found in the area, they were recruited from other areas of the city. The experts were contacted by the researchers in each country via telephone or email and invited to participate in the study, the purpose of which was fully explained to them. All interviews were carried out face-to-face and audio-taped.

The interviewees were employees in a wide range of services in the not-for-profit and state sector and had a variety of professional backgrounds: Social workers (8), Psychiatrists (7), Psychologists (5), Educators (2) Psychiatric nurse (1), Medical Doctor (1), Lawyer (1), Nurse (1), Homeless service manager (1) & Therapist (1).

The semi-structured interview was developed using an iterative process involving all partners and translated into the languages of all participating countries. One pilot interview was conducted in each participating country. The overall interview protocol consisted of (i) two case vignettes which described two patients with different mental health problems and with different attitudes towards seeking care. The experts were asked about their pathways into care, the barriers to receiving care and ways to overcome these barriers. The same vignettes were used across all capitals to ensure consistency; and (ii) four questions regarding the strengths, weaknesses and co-ordination of services for homeless people with mental health problems, and how service provision may be improved.

For the purposes of this paper the research questions analysed were a) what are the barriers to mental health care for homeless people? b) what are the ways to overcome these barriers? c) what are the two most important changes in practice that would improve care for homeless people with mental health problems?

All interviews were transcribed, ensuring the removal of any identifying information to maintain anonymity, and were translated into English. The study coordinating centre examined the translated transcripts and sought any necessary clarifications from the respective centres.

Ethical approval was not required in the participating countries for this study, as there was no health intervention and no personal information was collected.

### Data analysis

PASW 18.0 was used to analyse the assessment of services data. Chi-Square and Mann–Whitney tests were used to assess any significant differences in service provision between the homeless specific services and the generic services assessed as part of the wider project. All statistical tests were two-tailed and the significance level was set at (p<0.05).

The semi-structured interview transcripts were analyzed using thematic analysis [14]. The data from the initial 12 transcripts were coded independently by researchers from two capital cities and a coding frame was produced. This coding frame was then used to code the remaining 16 transcripts. This was carried out by one researcher at the project co-ordinating centre. The initial codes were then merged into categories and further refined into conceptual themes. [15,16]. The frequency counts of themes and the corresponding categories were recorded. The emerging themes were regularly revised at the co-ordinating centre and discussed within the wider international project group.

## Results

### Assessment of services

**Figure 1:**
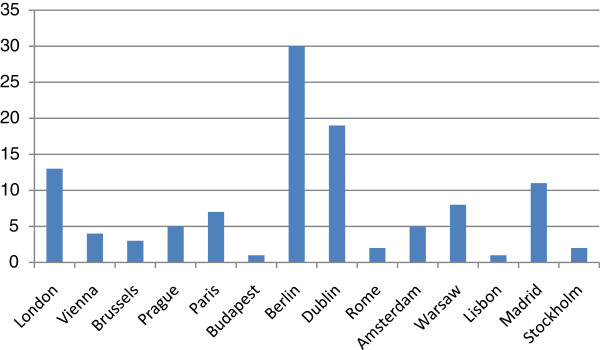
Number of services assessed in each capital city (median = 7, SD = 8.29).

In total 111 homeless-specific services were assessed. As can be seen in Figure 1, there was a high level of variability in the number of services assessed across the participating capital cities.

Nineteen of the 111 services assessed were described as homeless-specific mental health services. These services were found in seven of the 14 participating capital cities: Berlin (5), Paris (4), Dublin (3), London (3), Stockholm (2), Amsterdam (1) and Rome (1). Eighty-four services were described as homeless-specific social care services and were found in all participating capital cities bar one. These services included 30 accommodation related services, 17 day centres, 13 social support services providing for example advice and support, and 5 outreach services. The remaining 19 services provided more than one service type e.g., accommodation plus outreach. Eight services were described as homeless-specific general health services, all of which were primary care services. These services were found in four participating capital cities: Berlin (4), London (2), Dublin (1) and Warsaw (1).

A summary of the main findings relating to service provision in the research areas of the 14 participating capital cities is shown in Table 2.

**Table 2:** Characteristics of assessed services across all research areas in participating capital cities (n = 111)

**Variable**	**N%**	**Variable**	**N (%)**
**Accessibility**		**Programmes provided**	
Accepting self-referrals	89 (80.2%)	Active outreach	42 (37.8%)
Open outside office hours Mon-Fri	58 (52.3%)	Case finding	30 (27%)
Open and time at weekends	67 (60.4%)	Counselling	70 (63.1%)
Services requiring out of ‘pocket’ fee for payment	40 (36%)	Individual psychotherapy	18 (16.2%)
Waiting lists for any aspect of the service	31 (27.9%)	Detoxification treatment	12 (10.8%)
		Drug addiction treatment	15 (13.5%)
**Exclusion Criteria**		Alcohol addiction treatment	17 (15.3%)
Addiction	25 (22.5%)	Support around social welfare	85 (76.6%)
Aggressive behaviour	49 (44.1%)	Housing advice and support	95 (85.5%)
Criminal history	7 (6.3%)	Legal advice and support	59 (53.2%)
Command of language of host country	14 (12.6%)	Job coaching and finding	67 (60.4%)
Lack of motivation	28 (25.2%)	Mental health advocacy	44 (39.6%)
**Co-ordination**		**Staff Supervision**	
Routine meetings at least once a month concerning the care of homeless people	60 (54.1%)	Internal supervision	58 (52.3%)
		External Supervision	50 (45.5%)
**Evaluation: Systemic recording of**		**Services provided by**	
Socio-demographic characteristics of clients	97 (87.4%)	State	32 (26.1%)
Attendance and care provided	85 (76.6%)	Not for profit/private	79 (71.2%)
Clients satisfaction and experience	45 (40.5%)		

Twelve of the assessed services were provided specifically for women and 10 specifically for men. Overall 64% of services reported some form of exclusion criteria**,** with aggressive behaviour being the most prominent. There was a high level of variability across countries in terms of active outreach provision. The research areas in Paris and London reported the highest levels of outreach provision (71% and 62% respectively) with the lowest reported in Warsaw and Vienna (13% and 0% respectively). High levels of variability were also reported in the provision of internal supervision for staff and whether aggressive behaviour on the part of homeless people was a reason for exclusion from service.

**Figure 2:**
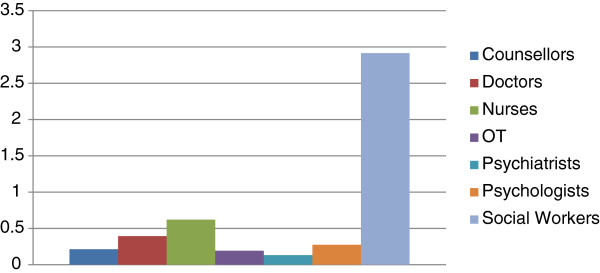
Average number of reported professional staff per service (whole time equivalent) (n = 111).

The median number of whole time equivalent (WTE) staff reported across all services was 7.25 (interquartile range 12.00). The percentage of services providing professionally qualified health care staff can be seen in Figure 2. The majority of services (70%) reported that they do not employ any professionally qualified mental health staff (a psychiatrist, a psychologist/psychotherapist or a counsellor to a combined WTE of at least 0.5). 29% reported that they do not employ social care staff (an occupational therapist or a social worker to a combined WTE of at least 0.5). In terms of peer support 9 (8%) of services reported that former clients are involved in direct delivery and contact with clients in a paid role and 16 (14%) in an unpaid role.

92% of services assessed reported providing some type of social care programme (social welfare support, housing support, legal advice and support or job coaching/finding). 21% of services reported that they provide some type of addiction treatment programme (detoxification treatments, drug addiction treatments or alcohol addiction treatments).

Of the 350 generic services assessed as part of the wider project, 148 (42.3%) reported that they document whether the client is homeless and 28 (8%) reported that they provide a specific programme for homeless people. In comparison to the generic services assessed, homeless-specific services (n = 111) were significantly more likely to be provided by ‘not for profit private organisations’ (71.2% vs 47.7%, p<0.01**); to engage in case finding (27.0% vs 16.9%, p<0.05*); to provide some type of social care programme (92.0% vs 76.1%**); and were less likely to report having a waiting list (27.9% vs 47.0%**).

On the other hand, the generic services assessed were significantly more likely to report providing addiction programmes (42.1% vs 20.9%**); individual psychotherapy (48.0% vs 16.2%**); and to have a higher number of paid staff (median 9.5 [interquartile range 21.5] vs 7.3 [12.0]*). They were also significantly more likely to report having doctors (26.7% vs 16.5%*); psychiatrists (34.9% vs 11.0%**); psychologists/psychotherapists (50.6% vs 22.0%**); and occupational therapists (22.9% vs 6.4% **) as part of staff. On the other hand the homeless specific services were significantly more likely to report having social workers (68.8% vs 57.2%*) as part of staff than the generic services assessed.

Services which were defined as homeless-specific mental health services (n = 19) were significantly more likely to report providing active outreach (63.2% vs 33.6%*) and support around housing (84.2% vs 58.9%*) than the generic mental health services assessed as part of the wider project (n = 221). However, they were significantly less likely to report having psychiatrists (21.1% vs 47.5%*), psychologists/psychotherapists (36.8% vs 60.2%*) and occupational therapists as part of their staff (5.3% vs 28.9%*), and to provide psychotherapy (5.3% vs 63.5%**).

### Barriers to care and ways to overcome them

The most common themes and corresponding categories arising in the interviews are presented here. The number of experts who highlighted the issue is indicated in each case (n = 28).

### Barriers

A common theme identified was the difficult and chaotic life circumstances of homeless people (23 experts), including alcohol and substance abuse issues (13) and difficulties in maintaining medication compliance (10). The unwillingness amongst the homeless population to engage with the services was also seen as a barrier (17), often due to a lack of trust in health professionals (12).

Barriers relating to health insurance were frequently reported (15), mainly relating to not having insurance or not being registered with a General Practitioner (GP) (14). Admission and discharge procedures in the health services were also highlighted (11), with the main barrier here being a lack of clear responsibility within the services in relation to the treatment of homeless people and complex rules in relation to catchment areas (8).

Lack of collaboration between mental health, social welfare and homeless services (14) was also highlighted frequently by the experts, as was a lack of mental health outreach provision (10)*.* Prejudice/negative responses by health professionals towards homeless people were regularly highlighted (15). Barriers linked to the provision of homeless accommodation (15) were underlined, with lack of capacity being the main issue (9).

### Ways to overcome barriers

Building a relationship of trust with clients (19) was frequently highlighted. Amongst the suggested ways of doing this were using an unintrusive approach (12), being respectful to clients (11) and ensuring regular contact (11). Addressing administrative barriers were referred to extensively (16), mainly in relation to helping homeless people to obtain health insurance and with admission procedures (12).

More collaboration between mental health, social welfare and homeless services was recommended by 18 experts. The importance of taking a comprehensive approach to treatment was alluded to by 16 experts, mainly in terms of helping clients in solving social and welfare issues (15) e.g. stable accommodation, employment. Greater provision of mental health outreach (11), particularly on the streets (9) was also highlighted.

### Improving service provision

Each expert (n = 28) was asked what two changes would most improve mental health care provision for homeless people in their area. The most common categories of response were:

· Improving collaboration between mental health and homeless services (n = 12)

· Providing specialised mental health teams and professionals to work with homeless people (n = 12)

· Providing mental health outreach on the street (n = 12)

· Assisting clients to solve housing issues (n = 12)

## Discussion

The PROMO study has highlighted a number of issues in relation to service provision for homeless people with mental health problems in Europe. Low levels of outreach and case finding were reported across the assessed services, out of hours service provision appeared inadequate and high levels of exclusion criteria were evident. The level of input from mental health professionals was generally reported as being low. Substance abuse was highlighted as a major issue amongst homeless people yet addiction programme provision is low. Prejudice in the services towards homeless people, a lack of co-ordination amongst services and difficulties homeless people face in obtaining health insurance were identified as major barriers to service provision.

The data suggest that homeless-specific services in Europe are currently more sympathetic to the initial needs of homeless people i.e. engagement with services. They are more likely to provide case finding, outreach and to provide support on issues such as housing and social welfare than generic services. The larger generic type services are more likely to provide addiction programmes and psychotherapy, to employ mental health professionals and have a multidisciplinary team and, therefore, may be more appropriate for long term care.

Homeless people in Western countries are more likely to have alcohol and drug problems than the general population [1]. It is estimated that between 20% and 50% of homeless people with mental health problems are also diagnosed with a substance abuse disorder [17-19], and find it difficult to access appropriate services. The issue of substance abuse amongst homeless people was highlighted by experts in this study, while almost a quarter of services assessed reported that they would exclude a homeless person with an addiction. Low levels of addiction programmes were reported in the assessed services, which corresponds to what has previously been reported in the literature [7,10,11].

In relation to out of hours service provision only 60.4% of services reported that they open sometime at weekends while just over half open outside normal office hours. This is similar to a study in the UK in 2002, which assessed health authorities with services for rough sleepers and found that only 55% had services that are open out of hours [7].

Staff with a professional qualification tend to be in the minority in organisations providing services for homeless people in a number of EU countries [12]. With the exception of social workers, low levels of professional health care staff were reported in this study, in particular mental health staff. The need for more mental health outreach was identified, particularly on the streets. Street outreach engages clients who are more severely impaired, are less motivated to seek treatment and take longer to engage with services [9]. Evidence suggests that assertive community treatment programmes are effective in treating homeless people with mental health problems in comparison to regular treatment [20,21].

Health insurance results in fewer barriers to accessing care, greater use of outpatient services and better compliance with medication amongst homeless people [22,23]. Barriers in relation to health insurance and the subsequent difficulties in obtaining services were identified in a number of participating countries. Helping homeless people to obtain health insurance and with the complex admission procedures they often face were highlighted as important aspects of improving access to care.

Co-ordinated treatment programmes, including case management, are more effective than usual care for homeless people [6]. This level of collaboration, however, is still the exception rather than the norm [10], and can prevent people from being linked into the services they need at the earliest opportunities [7]. In this study the general lack of collaboration between mental health, social welfare and homeless services was identified as a barrier to overall quality of service provision. Also, just over half of the services assessed reported that they have routine meetings at least once a month with other services concerning the care of homeless clients.

In many countries, non-governmental organisations (NGOs) have a tradition of providing services for homeless persons. In 2007 NGOs were the principal direct providers in seven of nine EU countries analysed [24]. This was also reflected in the current study where ‘not for profit private services’ i.e. NGOs, were the main providers. The degree of integration/coordination with state level provision of mainstream mental health services, therefore, emerges as an important service delivery issue.

### Limitations

In interpreting the findings from this study, it must be borne in mind that the assessment of services is based on reports from service staff in the research areas. Validation of responses, in terms of access to actual service data or the views of service users from the target groups, was beyond the remit of this study. All services included in the assessment of services were equally weighted in the analyses, with no consideration of their size or how many homeless people they serve.

The interpretation of some of the questions in the assessment of services questionnaire may have differed across capital cities. As an example, it is difficult to determine whether the term ‘active outreach’ was interpreted in the same manner in all participating countries. This may explain some of the variability in outreach provision reported across participating cities. The other variables which showed a high level of variability across cities, internal supervision and aggressive behaviour, may also have being open to different interpretations across countries.

There was much variability in the number of services assessed across the capital cities, with three cities contributing over 50% of the assessed services. While every effort was made to ensure consistency of recruitment across capital cities, this was difficult to ensure in such a large multi-centre study. Considering the differences in the overall provision of mental health care in general across countries in this study, it is not altogether surprising that fewer services are involved with providing some form of mental health care for homeless people in certain capital cities. Also, in some capital cities services relating to homeless people tend to me more centralised (e.g. Stockholm) and in other cities more dispersed (e.g. Dublin).

The variability in services assessed across cities may in part be due to the demographic profile of each research area. Taking the data from Dublin as an example, all of the homeless services assessed (n = 19) were located in one research area as this area incorporated an area of the inner city where homeless people tend to congregate. No homeless services were identified in the second research area. Research areas were identified in order to maximise the number of services catering for all assessed marginalised groups in the overall project, and not specifically homeless people.

The identification of two distinct marginalised areas with relatively large populations was in some ways more appropriate for the larger capital cities. In some of the smaller capital cities there was an overlap of research areas where in some cases a service identified was relevant to both research areas rather than being distinct to one. This may have reduced the numbers of services overall in the smaller capitals.

Considering these issues it is difficult to make comparisons on service provision across capital cities. However, the aim of the assessment of services was to give a general overview of service provision for homeless people with mental health problems in Europe. Considering that data was collected from 14 capital cities across Europe we felt it was important to include all homeless services assessed in the analysis in order to get the most complete overview. This was done while taking into consideration the variability in numbers of services assessed across cities, whether real or a result of other factors.

A major limitation is that the views of homeless people in receipt of services were not taken into account when assessing service provision and barriers to care. This was demeed to be outside the remit of the current study. It is also worth noting that services assessed in this study reported that they were less likely to collect information regarding client satisfaction and experience than on socio-demographic characteristics and attendance and care provided.

The assessment of services was focussed on homeless-specific services. As homeless people tend to access Accident and Emergency services regularly when seeking care it would be beneficial in the future to focus more specifically on service provision within Accident and Emergency services. While some Accident and Emergency services were assessed as part of the overall study, they were not assessed in sufficient numbers across countries to conduct a specific analysis and were therefore defined as generic general health services.

## Conclusions

The findings outlined in this study have a number of implications for health policies targeting homeless people with mental health problems in Europe. Notwithstanding the limitations outlined above the data indicates that service provision varies across European capital cities. More in-depth research comparing the quality of service provision and service outcomes would be useful in this instance. It also highlights the need for more coordinated models of service delivery across Europe. The results do show, however, that there is consistency across Europe in relation to the main barriers to care for homeless people with mental problems, and how service provision may be improved.

Further research regarding the relative effectiveness of different service delivery models would lead to more explicit policy recommendations concerning the role of specific services for homeless people, both compared to generic services but also in relation to a targeted mental health component. Such research would also assist in identifying the most efficient pathways from homeless specific services to the generic services. The integration of both specific and generic services requires high levels of co-ordination, which this study suggests do not currently exist*.*

Enabling services to act before mental health problems become severe and intractable has been identified as a crucial component of homelessness prevention [25]. More comprehensive strategies for the care and treatment of homeless people with mental health problems which address the issues highlighted in this paper are necessary. Targeted policy responses, including a commitment to adequate funding, along with improved structures for coordination and inter-agency working should be developed in order to improve service delivery across Europe.

## Competing interests

The authors declare that they have no competing interests.

## Authors’ contributions

All authors contributed to the design of the study and the data collection in their respective countries. RC carried out the analysis and interpretation of the data and drafted the paper. MB contributed to the interpretation of the data and drafting of the paper. SP made substantial contributions to the design of the study and contributed to the drafting of the paper. AM contributed to the analysis of the data and the drafting of the paper. MB, AM, TG, UK, JM, CS, AS and SP revised the manuscript critically for important intellectual content. All authors read and approved the final manuscript.

## Pre-publication history

The pre-publication history for this paper can be accessed here:

http://www.biomedcentral.com/1472-6963/12/222/prepub
